# Room-Temperature C–C σ-Bond Activation of Biphenylene Derivatives on Cu(111)

**DOI:** 10.1021/acs.jpclett.2c03346

**Published:** 2023-01-23

**Authors:** Jan Patrick Calupitan, Tao Wang, Alejandro Pérez Paz, Berta Álvarez, Alejandro Berdonces-Layunta, Paula Angulo-Portugal, Rodrigo Castrillo-Bodero, Frederik Schiller, Diego Peña, Martina Corso, Dolores Pérez, Dimas G. de Oteyza

**Affiliations:** † Centro de Fisica de Materiales CFM/MPC, CSIC-UPV/EHU, 20018 San Sebastián, Spain; ‡ Donostia International Physics Center, 20018 San Sebastián, Spain; § Department of Chemistry and Biochemistry, College of Science (COS), United Arab Emirates University (UAEU), 15551 Al Ain, UAE; ∥ Centro Singular de Investigación en Química Biolóxica e Materiais Moleculares (CiQUS) and Departamento de Química Orgánica, Universidade de Santiago de Compostela, 15782 Santiago de Compostela, Spain; ⊥ Nanomaterials and Nanotechnology Research Center (CINN), CSIC-UNIOVI-PA 33940 El Entrego, Spain

## Abstract

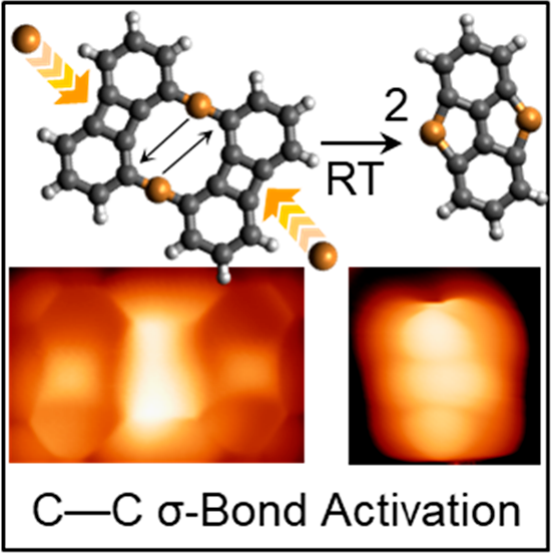

Activating the strong C–C σ-bond is a central problem in organic synthesis. Directly generating activated C centers by metalation of structures containing strained four-membered rings is one maneuver often employed in multistep syntheses. This usually requires high temperatures and/or precious transition metals. In this paper, we report an unprecedented C–C σ-bond activation at room temperature on Cu(111). By using bond-resolving scanning probe microscopy, we show the breaking of one of the C–C σ-bonds of a biphenylene derivative, followed by insertion of Cu from the substrate. Chemical characterization of the generated species was complemented by X-ray photoemission spectroscopy, and their reactivity was explained by density functional theory calculations. To gain further insight into this unique reactivity on other coinage metals, the reaction pathway on Ag(111) was also investigated and the results were compared with those on Cu(111). This study offers new synthetic routes that may be employed in the *in situ* generation of activated species for the on-surface synthesis of novel C-based nanostructures.

Metal-mediated activation of nonpolar σ-bonds is of central importance in synthetic chemistry,^1−9^ and therefore in a multitude of relevant industrial processes (e.g., pharmaceutical, polymer processing, cosmetics, and agriculture). Metalation allows for (i) the cleavage of otherwise strong C–C (or C–H) σ-bonds and (ii) the insertion of a transition metal (catalyst) to activate a C atom for further chemical transformations. Both C–H and C–C σ-bonds of organic structures may be metalated. Generally, the former is used during initial stages of multistep synthesis^2,3^ for eventual functionalizations (C–heteroatom bond formations) or C–C couplings, while the latter is usually employed for late-stage modifications of a preexisting carbon framework.^1,2,4−6^ An otherwise strong C–C bond is rendered susceptible to metalation by geometric and/or electronic directing effects. One strategy takes advantage of thermodynamically compromised C–C bonds in strained four-membered rings. Indeed, a rich chemistry has been developed around strained four-membered cyclic carbon structures, susceptible to oxidative addition to transition metal complexes that can lead to transformations such as ring opening, annulation, coupling, and (de)carbonylation, to name a few.^7,8,10,11^

The unique structure of biphenylene, with two benzene rings fused to a strained antiaromatic cyclobutadiene (Scheme 1), makes it an excellent candidate for C–C activation and further incorporation of the resulting 2,2′-biphenyl moiety into more complex polycyclic aromatic systems.^1,7,9,11^ Precious metals such as Au,^12−16^ Pt,^17,18^ Pd,^17,19,20^ Ir,^21−24^ and Rh^25−28^ have been shown to cleave and insert onto the biphenylene σ-bond, generating active species for a diverse set of reactions^7,11^ that includes dimerization,^18^ formal [4+2] or [4+1] cycloadditions,^18,23^ or coupling reactions.^19^ Although this has also been observed by using earth-abundant metals such as Fe,^29,30^ Co,^31^^32^ Ni,^33−36^ and Al^37^ to the best of our knowledge,^11^ biphenylene C–C bond activation has not yet been observed for Cu at room temperature. Further expanding such a process to more metals would provide alternatives to rare transition metal catalysts.^38^ In addition, high temperatures are usually required to break the C–C bond due to its strength.^13^ Although C–C σ-bond metalation has been observed at room temperature,^12,14^ achieving it with an earth-abundant metal such as Cu would open the field to more environmentally friendly metal catalysts, especially because the quasi-simultaneous cleavage and metal insertion into aryl C–C σ-bonds afford large atom economies^10,11^ in multistep synthesis.

**Scheme 1:**
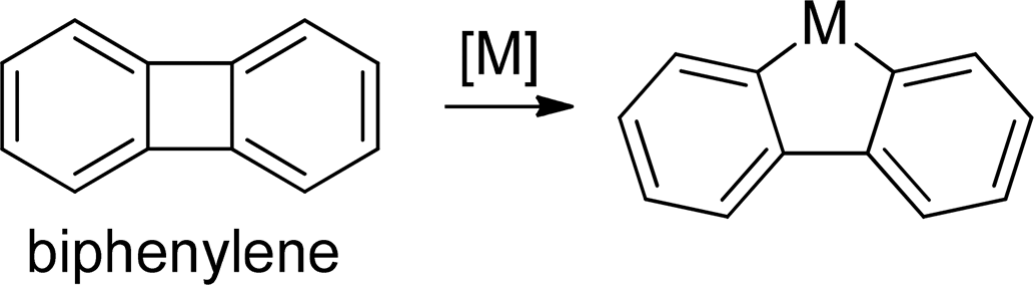
C–C Bond Activation of Biphenylene (C_12_H_8_) Has Been Reported on Several Metals (M = Au, Pt, Pd, Ir, Rh, Fe, Co, Ni, or Al)

Meanwhile, in the context of nanoscience, biphenylene remains largely untapped as a precursor in the blooming field of on-surface synthesis^39−43^ (OSS) despite its rich solution-phase chemistry described above. OSS has produced and characterized graphenic^41^ nanostructures with the aid of state-of-the-art scanning probe techniques. OSS commonly proceeds by Ullmann coupling between halogenated phenyl moieties at increased temperatures. In a nutshell, precisely designed precursors, upon deposition onto metal surfaces, are substituted with surface adatoms before coupling and subsequent aromatization. This remains the most common route for OSS of polyaromatic structures, so new synthetic routes need to be explored. Although on-surface C–H σ-bond activation^42−45^ and surface-mediated reactions involving C–C bond breakage^46^ have been reported, these required temperatures above room temperature (RT). In addition, the quasi-simultaneous C–C bond breakage and metal insertion remain hardly explored on surface. Direct production of a reactive synthon via insertion of a metal into a C–C σ-bond could open up new synthetic strategies for the construction of C frameworks on surface.

In this work, we report the unprecedented activation of a C–C σ-bond on Cu(111) at room temperature under UHV conditions, as observed by low-temperature scanning tunnelling microscopy (LT-STM). In particular, we explore the ring-opening reaction of a dibrominated biphenylene derivative [1,8-dibromobiphenylene, henceforth **1** (Scheme 2)] on Cu(111) and compare its reactivity to that on Ag(111). On both surfaces, the organometallic dimers **2** are observed readily at RT. On Ag(111), subsequent annealing transforms organometallic dimer **2-Ag** into product **3**. Meanwhile, on Cu(111), **2-Cu** readily evolves at RT via metalation of a C–C σ-bond. The resulting species **4** transforms into nanographene fragments upon being further heated. The results are supported by X-ray photoemission spectroscopy (XPS), and the differences in reactivity between the two metal surfaces are explained by density functional theory (DFT) calculations.

**Scheme 2:**
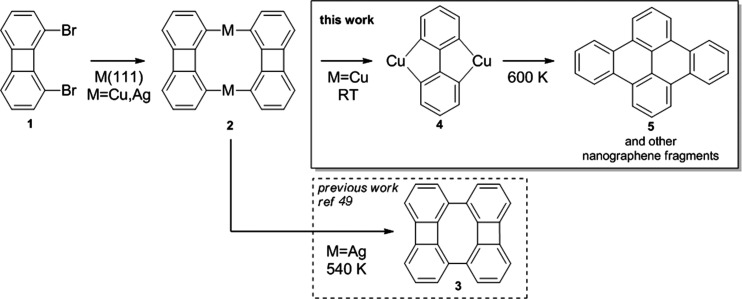
On-Surface Products Obtained from Deposition of a 1,8-Dibromobiphenylene Precursor (**1**) on Different Surfaces C–C bond activation occurs on Cu(111), while a double Ullmann coupling reaction occurs on Ag(111).

The synthesis of 1,8-dibromobiphenylene (**1**) has been performed following previously reported procedures with minor modifications^47,48^ (see the Supporting Information for details). Deposition of **1** on Ag(111) and Cu(111) kept at approximately 270 K leads to spontaneous C–Br homolytic bond cleavage and results in different structures. On Ag(111), **1** forms organometallic dimer **2-Ag** (Figure S1). In line with a previous work in which **2-Ag** was obtained from a different tetrabromobiphenyl precursor, it transforms into its (double) Ullmann coupling product **3** upon annealing.^49^ Meanwhile, on Cu(111) the deposition of **1** results in two products (Figure 1a). The major product is a rectangular molecule with a bright protrusion in the center that looks similar to **2-Ag** (Figure 1a, yellow arrows), while the minor product appears thinner and relatively higher and shows a stronger protrusion (Figure 1a, white arrows). Bringing the sample to room temperature results in the transformation of all of the rectangular species into the thinner ones (Figure 1b). High-resolution STM imaging using a CO-functionalized tip has been used to assign the structures of both products. Figure 1c shows the bond-resolving image of **2-Cu** (see Figure S2 for the STM image superimposed with the structure of **2-Cu**). In analogy to **2-Ag**, the two bright protrusions in the center correspond to two Cu atoms. Figure 1d shows a high-resolution image of the products in Figure 1b, attributed to **4**, formed by two phenyl rings with vertices oriented parallel to the long molecular axis (see Figure S3a for the STM image superimposed with the structure of **4**). Organometallic dimers **2-Ag** and **2-Cu** therefore display different reactivities on their respective substrates. **2-Ag** is further transformed upon being annealed to 540 K, undergoing a demetalation to form **3** while the two four-membered rings are kept intact, whereas **2-Cu** readily reacts at RT by cleaving the four-membered ring and forming two extra C–Cu bonds forming **4**.

**Figure 1:**
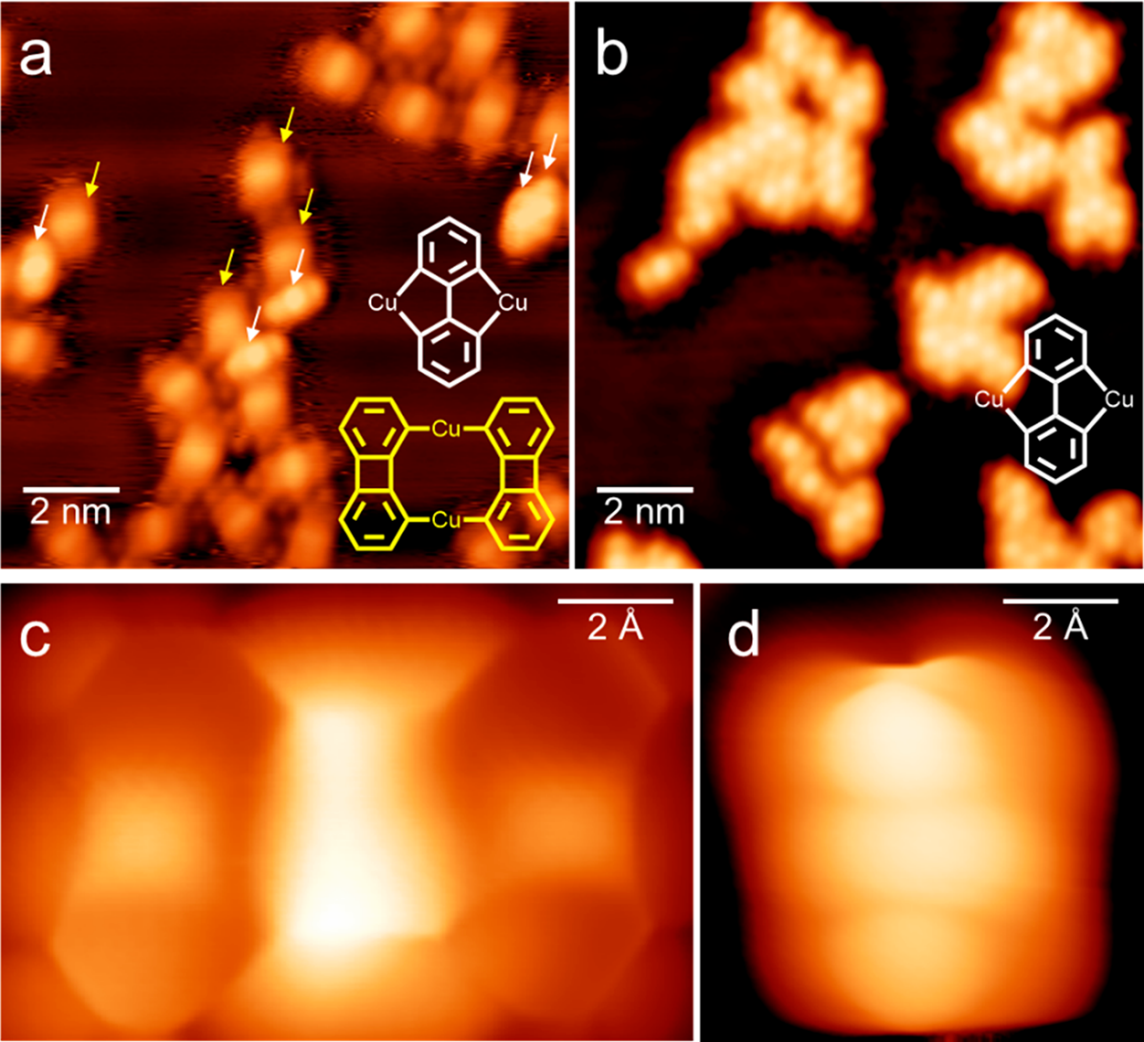
(a) Large scale STM images upon deposition of **1** on Cu(111) held at 270 K, showing mostly **2-Cu** products (yellow arrows) and some dicopper compounds **4** (white arrows) (imaging conditions: *U* = −700 mV, *I* = 300 pA, 78 K). (b) STM images after the sample had been annealed to room temperature (*U* = −500 mV, *I* = 1 nA, 78 K). (c) Constant-height high-resolution image of organometallic dimer **2-Cu** on Cu(111) with a CO-terminated tip (*U* = 5 mV, 4.3 K). We note the similarity in shape to the corresponding organometallic dimer **2-Ag** (Figure S1b).^49^ (d) Constant-current high-resolution image of **4** with a CO-terminated tip (*U* = −300 mV, *I* = 1.5 nA, 5 K).

Further annealing of **4** to 600 K results in flat structures (Figure 2a and Figure S4) intermixed with unreacted **4** (black arrows). The Br atoms remain on the surface, visible as spherical protrusions around the flat structures, and drive the intermolecular interactions and molecular aggregation.^50^ XPS measurements of the Br 3p core levels reveal unchanged binding energy and intensity (Figure S5). Bond-resolving STM images of the flat structures reveal laterally coupled biphenyl units (Figure 2b). These results indicate the removal of Cu atoms from **4**, generating biphenyl units. This is in line with previous studies that report both the removal of Cu atoms from organometallic structures and the subsequent C–C coupling at ≥375 K.^51−53^ We note that it is also possible to find biphenyl moieties, i.e., after Cu removal, connected to other phenyl rings in some regions of the sample (Figure S4b).

**Figure 2:**
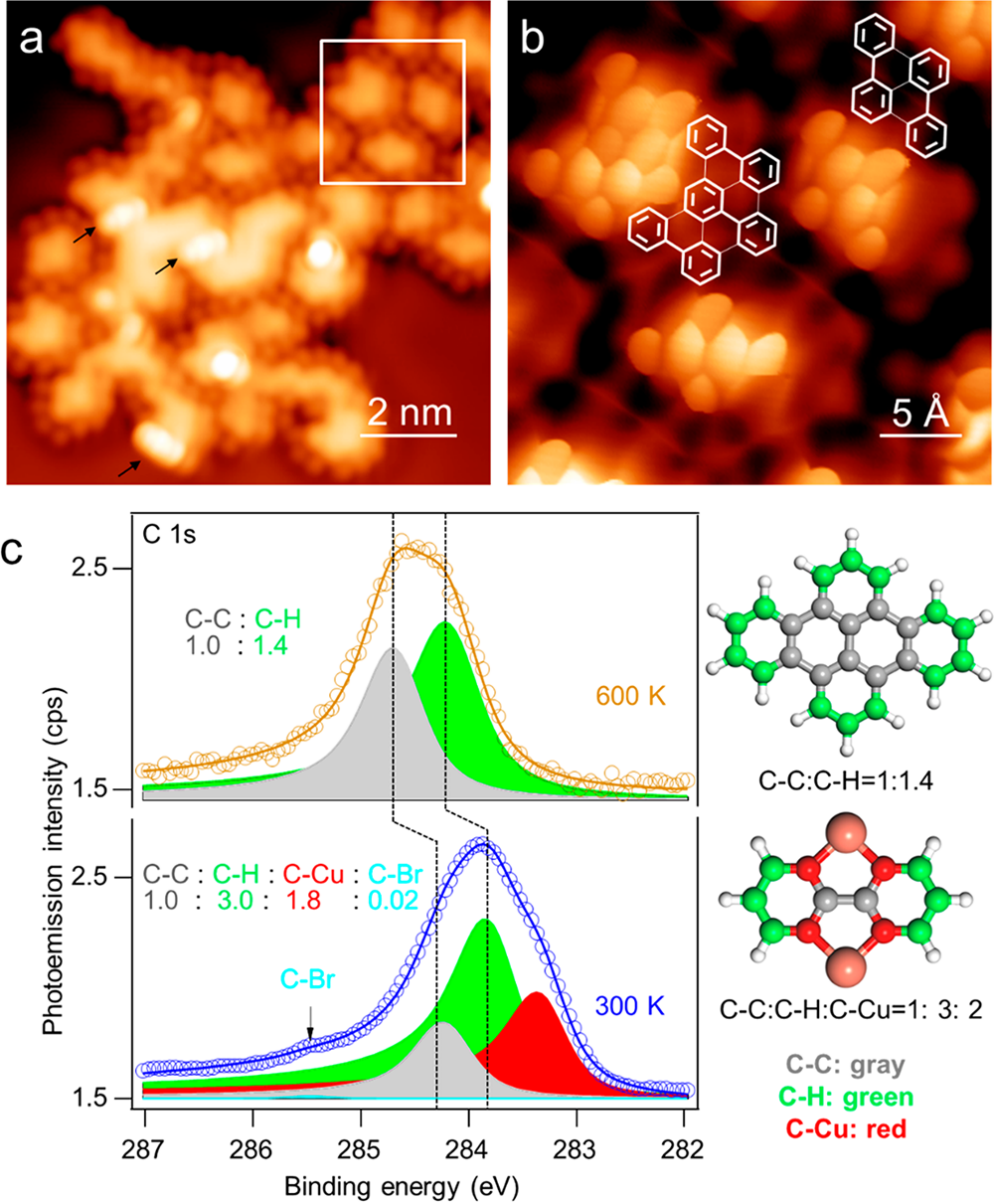
(a) STM images upon annealing at 575 K. Black arrows point to unreacted **4** (*U* = −500 mV, *I* = 100 pA, 4.3 K). (b) High-resolution bond-resolving STM image of the region by a white square in panel a. Chemical structures are drawn with the biphenyl units highlighted for reference. (c) XPS analysis of **4** and its post-annealing products.

X-ray photoelectron spectroscopy (XPS) analysis of **4** and **5** supports their structural assignments. Figure 2c shows the C 1s core level of a sample containing **4** before and after annealing to obtain **5**. Compound **4** contains three chemically distinct sp^2^-hybridized C atoms: two surrounded by other C atoms, four connected to the Cu atoms, and six attached to one H atom. Deconvolution of the C 1s signal results in three main peaks whose integration ratios are in good agreement with the stoichiometry of distinct C species in the molecule. We note that the increasing binding energy of C atoms connected to Cu, H, and other C atoms is consistent with the decreasing electron density due to their relative electronegativities. In other words, carbon atoms bound to other less electronegative atoms will normally display a higher electron density and therefore lower binding energies.^54,55^ After annealing, only two components are necessary to obtain a good fit. Their integrated intensities agree with the ratio of two distinct C atoms in the molecule: 10 C(−C) atoms and 14 C(−H) atoms, consistent with the demetalation. Both components appear to be rigidly shifted by ∼0.4 eV to higher binding energies, presumably as a result of the absent metal coordination-related charge transfer to the molecule upon demetalation. Indeed, shifts of a similar magnitude and a similar direction have been predicted for other hydrocarbon structures when changing from a metal-coordinated to a noncoordinated form.^54^

To determine whether the formation of **2-Cu** is a necessary condition for the opening of the four-membered ring, a control experiment was performed with the nonbrominated counterpart of precursor **1**, biphenylene (C_12_H_8_). It was found that the absence of cleavable C–Br bonds in the precursor precludes the generation of **2-Cu** and confirms that the electronic activation of the neighboring bonds around the four-membered ring is required for σ-bond cleavage. Large scale images (Figure 3a) show that only one species is observed, and bond-resolving images with a CO tip (Figure 3b) confirm that the four-membered ring in biphenylene is intact on Cu(111). Furthermore, the sample remains stable and unchanged after several days at room temperature (Figure S6), indicating that the intrinsic thermodynamic instability of the four-membered ring, due to ring strain and antiaromatic character,^7,8,10^ is not enough to spontaneously cleave it on Cu(111) at room temperature. Instead, prior activation of the C–Br bonds (as in **2-Cu**) is necessary to trigger C–C σ-bond breaking at room temperature.

**Figure 3:**
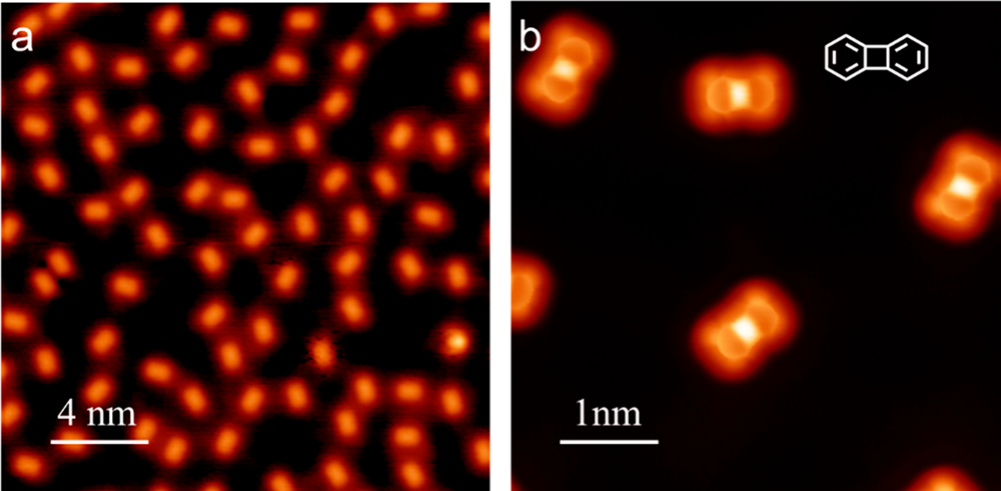
(a) Large scale STM image of biphenylene (C_12_H_8_) (*U* = 1 V, *I* = 50 pA, 4.3 K). (b) High-resolution bond-resolving STM image of biphenylene taken with a CO-terminated tip.

We have also performed DFT calculations to shed light on the difference in the reactivities of the four-membered rings of **2-M** on Cu(111) and Ag(111) (see the Supporting Information for computational details). Figure 4a summarizes the reaction pathway energetics on each surface starting from intermediate **2-M**. At first glance, the two pathways differ by how metal atoms react with **2**. While pathway I removes two metal atoms, pathway II requires an input of two metal atoms. Given that the activation energy of Cu adatom diffusion on Cu(111) is less than half (0.026 eV) of that of Ag on Ag(111) (0.059 eV),^56^ pathway II is preferred on Cu(111), where there is a larger supply of mobile Cu atoms that may participate in the reaction. On the contrary, pathway I is preferred on Ag(111) due to the weaker Ag–C bond. The calculated Ag–C (2.12 Å) bond length is longer than Cu–C (2.02 Å), and more importantly, the bond energy of the former is lower than that of the latter, facilitating the demetalation on **2-Ag**. To better compare the relative strengths of M–C bonds (M = Ag or Cu) in **2-M**, we have calculated the reaction energies of the homolytic bond cleavage of **2-M** into two mono-metalated biphenylene units (C_12_H_6_M) in the gas phase. We define the binding energy (BE) between two units held by different metals as BE = 2*E***(C_12_H_6_M)** – *E*_**2-M**_ [where M = Ag or Cu (see Figure S7)]. Higher BEs correlate with stronger bonds. In the gas phase, PBE0+D4/def2-TZVP calculations yield BEs of 427 kJ/mol (4.42 eV) and 388 kJ/mol (4.03 eV) for **2-Cu** and **2-Ag**, respectively, confirming the stronger character of M–C bonds on **2-Cu** than on **2-Ag**. This is in agreement with exhaustive *ab initio* DFT calculations by Rijs et al. that show dissociation energies of organosilver bonds are lower than those of organocopper bonds by ∼50 kJ/mol for various organic substituents.^57^

**Figure 4:**
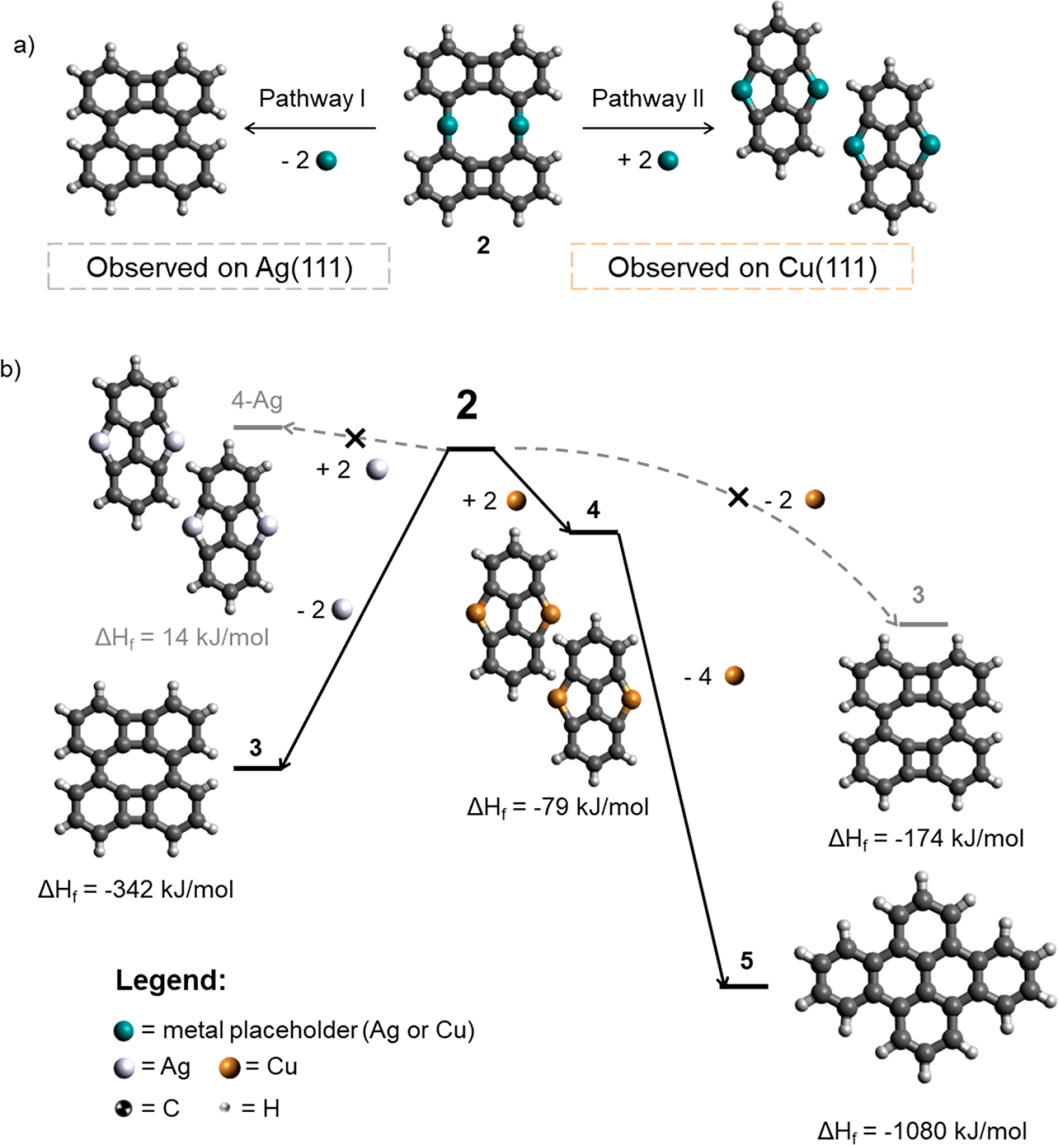
Schematic diagram of (a) the two reactions observed on Ag(111) and Cu(111) and (b) calculated relative gas-phase energetics from DFT. Legend: cyan, placeholder for metal centers (Ag or Cu); white, Ag; brown, Cu; black, C; white, H.

In addition, Figure 4b displays an energy diagram with the calculated energetics (Δ*E*) of molecules **3**–**5** relative to their respective organometallic dimer precursors **2-Ag** and **2-Cu**. The corresponding hypothetical organometallic silver complex of **4** (**4-Ag**) has been also included in the calculations. In the gas phase, the formation of **4-Ag** is found to be mildly endothermic (Δ*E* = 14 kJ/mol = 0.145 eV) while **4** is found to be exothermic (Δ*E* = −79 kJ/mol = −0.819 eV), which explains why **4-Ag** is not observed on Ag(111). Instead, annealing to 475 K directly transforms **2-Ag** into Ullmann coupling product **3** in a highly exothermic demetalation process (Δ*E* = −342 kJ/mol = −3.54 eV). On Cu(111), the formation of **3** (not observed) from **2-Cu** is less exothermic (Δ*E* = −177 kJ/mol = −1.83 eV) and therefore expected to require surmounting an energy barrier higher than that on Ag(111), requiring annealing to >475 K.^39−41^ This is consistent with the Bell–Evans–Polanyi principle, which states that for a family of related reactions, exothermicity correlates with a lower activation energy.^58,59^ Thus, the thermal demetalation of **2-Ag** should proceed faster than that of **2-Cu**.

At temperatures lower than 475 K, opening of the four-membered ring on **2-Cu** to form **4** proceeds via an exothermic process with a Δ*E* of −79 kJ/mol (−0.82 eV). This energy landscape hinders the formation of **3** from **2-Cu**; annealing to higher temperatures would simply push the system to overcome the lower barrier toward the formation of **4**. From **4**, further annealing results in the removal of the Cu atoms toward the formation of polyaromatic hydrocarbons such as **5** (Δ*E* = −1080 kJ/mol = −11.2 eV) and other fragments, well-known to be highly exothermic. Note that, although going from two units of **4** to **5** requires two additional hydrogen atoms, these are known to be available, even in their activated atomic form, from the residual H gas in ultra-high-vacuum chambers (especially in the presence of running filaments like those of ion gauges)^60^ or other dehydrogenative reactions occurring at high temperatures.

Although the elucidation of the reaction mechanism and accurate determination of activation energies are beyond the scope of this short communication, we attempt to propose a likely scenario for the opening of the four-membered ring in **2-Cu**, i.e., activation of the C–C σ-bond. DFT calculations (Figure S8) show that, on Cu(111), relaxed **2-Cu** has its sides (phenyl moieties) bent upward (Figure S8b). Thus, there is space (Figures S8b and S9) for mobile species (such as Br or Cu adatoms) to slide underneath and interact with the C–C σ-bond, thermally activating the bond (vibrations) toward cleavage. The two Cu atoms on the dimer rearrange in a metathesis reaction (Figure S8, gray arrows), probably in a synergistic or concerted fashion, giving **4** and its monometalated derivative (Figure S9). DFT calculations show that this monometalated product is 73 kJ/mol (0.76 eV) less stable than **4**, with the radicals at positions C-5 and C-5′ stabilized by the top layer of the Cu(111). Therefore, such instability gives it a thermodynamic driving force to capture another mobile Cu on surface (or lift another one from the top layer) to produce another unit of **4**.

To conclude, we show the C–C σ-bond activation of a dibrominated biphenylene derivative on Cu(111) at room temperature. Deposition of the precursor on Cu(111) produces an organometallic dimer that is metalated further by two additional Cu adatoms upon annealing to room temperature. The generated species further reacts toward polyaromatic hydrocarbons on surface at higher temperatures. XPS confirms the metalated structure at RT and the demetalation at higher temperatures (600 K). Control experiments show that the bromine atoms on the precursor and the formation of **2-Cu** are essential for the 4-membered ring opening to occur on Cu(111). DFT calculations give thermodynamic insight into the differences in the reactivity of the precursor on Cu(111) and Ag(111). Lastly, we have shown also how the activated organometallic compound can be used as an intermediate for the synthesis of polyaromatic hydrocarbons, offering new on-surface synthetic routes for the generation of novel C-based nanostructures.

## Supporting Information Available

Additional experimental details of the synthetic methods, STM and XPS experiments, and DFT calculations; supplementary STM images of the metal–organic dimers on Ag(111), the metal–organic dimer and monomer on Cu(111) with superimposed chemical structure drawings, resulting structures after high-temperature annealing on Cu(111), and pristine biphenylene on Cu(111); XPS spectra of the Br 3d core levels; and illustrations of reaction mechanisms and the DFT-optimized geometry of **2-Cu** on Cu(111) (PDF)
