# Supplementary material for: Room-Temperature C–C σ-Bond Activation of Biphenylene Derivatives on Cu(111)
Source: J Phys Chem Lett. 2023 Jan 23;14(4):947–53. doi: 10.1021/acs.jpclett.2c03346 (PMC9900639; doi:10.1021/acs.jpclett.2c03346)
Supplement: Supplementary file 1 — jz2c03346_si_001.pdf [file jz2c03346_si_001.pdf]

Supporting Information

Room-Temperature C—C σ-Bond Activation of Biphenylene Derivatives on Cu(111)

Jan Patrick Calupitan,[†,├] *Tao Wang,*[†,‡,├] *Alejandro Pérez Paz,*[§,├,*] *Berta Álvarez,*[‖] *Alejandro Berdonces-Layunta,*[†,‡] *Paula Angulo-Portugal,*[†] *Rodrigo Castillo-Bodero,*[†] *Frederik Schiller,*[†,‡] *Diego Peña,*[‖] *Martina Corso,*[†,‡] *Dolores Pérez,*[‖,*] *Dimas G. de Oteyza,*[†,‡,⊥,*]

[†]Centro de Fisica de Materiales CFM/MPC, CSIC-UPV/EHU, 20018 San Sebastián, Spain

[‡]Donostia International Physics Center, 20018 San Sebastián, Spain

[§]Department of Chemistry and Biochemistry, College of Science (COS), United Arab Emirates University (UAEU), PO Box: 15551, Al Ain, UAE

[‖]Centro Singular de Investigación en Química Biolóxica e Materiais Moleculares (CiQUS) and Departamento de Química Orgánica, Universidade de Santiago de Compostela, 15782 Santiago de Compostela, Spain

⊥Nanomaterials and Nanotechnology Research Center (CINN), CSIC-UNIOVI-PA; 33940 El Entrego, Spain

├ These authors contributed equally

METHODS

General synthetic methods (in solution)

Reactions were carried out under argon using oven-dried glassware. THF and Et_2O were purified by a MBraun SPS-800 Solvent Purification System. Other commercial reagents were purchased from Sigma-Aldrich or TCI, and were used without further purification. TLC was performed on Merck silica gel 60 F_{254} and chromatograms were visualized with UV light (254 and 360 nm). Column chromatography was performed on Merck silica gel 60 (ASTM 230-400 mesh). NMR spectra were recorded at 300 MHz (Varian Mercury-300 instrument).

Synthesis of 1,8-dibromobiphenylene (1)

1,8-Dibromobiphenylene was prepared in two steps from commercially available 1,3-dibromo-2-iodobenzene (**I**) following, with minor modifications, previously reported procedures:[12,13]

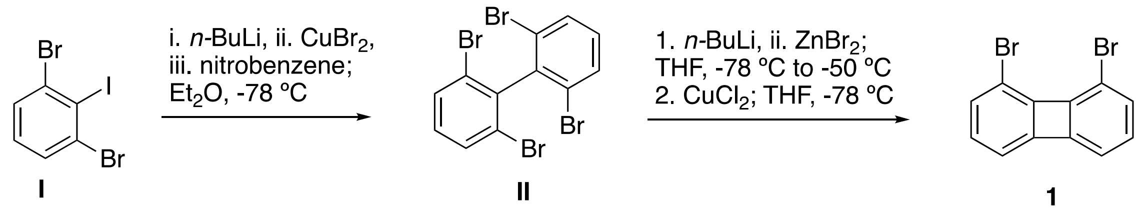

2,2',6,6'-Tetrabromobiphenyl (II)[13]

A solution of *n*BuLi (2.3 M in hexane, 3.6 mL, 8.29 mmol) was added to a stirred solution of 1,3-dibromo-2-iodobenzene (**I**; 3.00 g, 8.29 mmol) in Et_2O (16.6 mL) at -78 ºC under argon. Once the addition was completed, it was added copper(II)bromide (1.85 g, 8.29 mmol) and, after 45 min, nitrobenzene (3.4 mL, 4.08 g, 33.17 mmol). The reaction mixture was allowed to slowly reach room temperature over a period of 12 h. The resulting suspension was poured into a 12%

aqueous NH_4OH (15 mL). The organic layer was separated and the aqueous phase was extracted with ethyl acetate (2 x 20 mL). The combined organic layers were dried and concentrated under reduced pressure. From the resulting suspension, nitrobenzene was removed by careful decantation, and the solid was washed with cold hexane (2 x 20 mL) and dried under vacuum, to afford 2,2',6,6'-tetrabromobiphenyl (**II**) (740 mg, 38%). ^{1}H NMR (300 MHz, $CDCl_3$), d: 7.67 (d, *J* = 8.2 Hz, 4 H), 7.17 (t, *J* = 8.2 Hz, 2 H).

1,8-Dibromobiphenylene (1)

A solution of *n*BuLi (2.4 M in hexane, 0.675 mL, 1.62 mmol) was added dropwise to a solution of 2,2',6,6'-tetrabromobiphenyl (300 mg, 0.639 mmol) in THF (13 mL) at -78 ° C. After stirring the mixture for 2 h at -78 ° C, a solution of $ZnBr_2$ (158 mg, 0.702 mmol) in THF (2.0 mL) was added dropwise via cannula. The flask was transferred to a bath at -50 ° C, stirring was kept at this temperature for 2 h and, then, the solution was cooled to -78 ° C and cannula-transferred to a flask containing a suspension of anhydrous $CuCl_2$ (266 mg, 1.98 mmol) in THF (16 mL) at -78 ° C. The suspension was stirred for 1 h at -78 ° C and allowed to warm to room temperature. Then, aq. HCl (3 M, 20 mL) was added and the resulting solution was stirred for 10 min. The solution was extracted with benzene (3 x 40 mL), the extracts were combined and dried over $MgSO_4$, and the solvent was removed under vacuum. The crude product was purified by column chromatography (SiO_2, hexane) to yield 1,8-dibromobiphenylene (**1**) as a yellow solid (95 mg, 48%). ^{1}H NMR (300 MHz, $CDCl_3$), d: 6.84 (d, *J* = 8.3Hz, 2 H), 6.65 (dd, *J* = 8.3, 7.0 Hz, 2 H), 6.58 (d, *J* = 7.0 Hz, 2 H).

STM Experiments

STM measurements were performed using a commercial Scienta-Omicron LT-STM at 4.3 K. The system consists of a preparation chamber with a typical pressure in the low 10^{-10} mbar regime and a STM chamber with a pressure in the 10^{-11} mbar range. The Cu(111) and Ag(111) crystals were cleaned *via* two cycles of Ar^+ sputtering and annealing (720 K for Cu and 700 K for Ag). All STM and STS measurements were performed at either at 78 K or 4.3 K. To obtain BR-STM images, the tip was functionalized with a CO molecule that was picked up from the metal surfaces. CO was deposited onto the sample *via* a leak valve at a pressure of approximately 5×10^{-9} mbar and a maximum sample temperature of 7.0 K. CO can be picked up with a metallic tip by scanning with a high current and negative bias (*e.g.* I=1 nA, U=−0.5 V). dI/dV measurements were recorded with the internal lock-in of the system. The oscillation frequency used in experiments is 797 Hz.

1,8-Dibromobiphenylene **1** evaporated readily at room temperature at the working pressure of the preparation chamber (~10^{-10} mbar). The clean Cu(111) sample was initially measured by STM at the cryogenic temperature (4.3 K or 78 K) and was then transferred to the manipulator for depositing molecules atop. Then we opened the gate valve between preparation chamber and molecular evaporator (a home-built Knudsen cell) for 1 minute to deposit molecule **1** on Cu(111). Upon deposition, the sample was immediately returned to the STM head kept at the cryogenic temperature. This sample transfer process took approximately 7 minutes in total, during which the samples warmed up only to about 270 K before being put back into the cryogenic STM environment. For the room temperature annealing experiment, the sample was

taken out from the cold STM head and kept at the sample storage for a few hours. A similar procedure was used for the control experiment involving the biphenylene molecule whose evaporation temperature is 350 K and deposition time is 2 minutes.

XPS Experiments

X-ray photoelectron spectroscopy has been carried out holding the sample at room temperature and illuminating it with monochromatized Al Ka light from a SPECS μ-FOCUS 600 setup. The excited photoelectrons were collected by a SPECS 150 analyzer at an emission angle of 40°. The sample was first checked for possible contaminations that were not detected. Then the C 1s, Br 3p, and Cu 2p (not shown) spectral regions were investigated. C 1s and Br 3p core levels were further analyzed by peak fitting procedure. For this purpose, we used Doniach-Sunjik line-shapes[1] with Shirley background functions. Since individual width, asymmetry and background are unknown, we used constrains to limit to similar values allowing only a small variation. This resulted in the case of C 1s core levels to a width of (0.2±0.02) meV for 300K and (0.25±0.02) meV for 600K. The asymmetry value resulted in (0.18±0.02) and (0.12±0.02) for 300K and 600K, respectively. No appreciable background has been observed in the fits. The total intensity of the C1s peak is nearly identical before and after annealing to 600K. This means that no desorption of carbon atoms is detected. For the Br 3p core level, the fit gave a spin orbit splitting between the $3p_{3/2}$ and $3p_{1/2}$ components of 6.7 eV and an intensity ratio of 3:1. The leading $3p_{3/2}$ line was detected at 182.0 eV binding energy. Like for C 1s also in Br 3p no intensity drop after annealing was observed. Differences in the noise level are related to different acquisition times

(as in the C 1s spectra), but the intensities in counts per second remain similar, implying that the isolated Br stays on the surface rather than desorbing from it.

Computational Details of DFT Calculations

All slab density functional theory (DFT) calculations used the PBE exchange-correlation functional[2]. Since some molecules are physisorbed, we included van der Waals (vdW) corrections via the Grimme's D3 method[3] in all of our calculations.

The Cu(111) and Ag(111) surfaces were modeled with Cartesian coordinates derived from the experimental lattice constant of 3.615 and 4.090 angstroms, respectively, of the bulk metals keeping all metal atoms frozen except those of the top layer. Thus, only the adsorbate atoms and the metal atoms of the top layer were allowed to move during geometry relaxations. We carried out structural relaxations until a maximum force ~0.02~eV/angstrom was obtained on all atoms.

To investigate the adsorption and reactivity of the monomers we built a 5x5x4 Cu(111) slab (100 metal atoms, 25 metal atoms per layer) that features a minimum lateral adsorbate separation of over 4 angstroms between the closest atoms.

For the dimers, we used an enlarged 6x6x4 slab (144 metal atoms, 36 atoms per layer).

All initial geometries were constructed using the ASE module (version 3.21.1).[4] Over 15 angstroms of vacuum layer and dipole corrections were used to decouple the periodic images along the normal z direction.

We performed the slab calculations within the projector-augmented wave method (PAW)[5] as implemented in the code GPAW (version 21.1.0).[6,7] For the GPAW calculations, we used the plane wave (PW) mode with a converged kinetic energy cutoff of 500~eV. We used an

electronic Fermi-Dirac smearing temperature of 0.1 eV, and a reciprocal space K-point mesh of 3x3x1. With this PW computational setup, already used in Ref. [8], we reproduced the nearly bidegenerate Shockley surface state of ~0.55eV below the Fermi level at the Gamma (Γ) point for a clean unreconstructed Cu(111) slab and the experimental work function of 4.79 eV.

All gas-phase DFT calculations were performed using the program ORCA (version 4.2.1).[9] All geometry relaxations used the hybrid exchange-correlation functional PBE0,[10] supplemented with van der Waals corrections according to the Grimme's D4 approach[11]. We used the triple-zeta polarized basis set (def2-TZVP) from Ahlrichs and coworkers[12] supplemented by the auxiliary basis: def2/J[13]. We used "TightSCF" keyword for all geometry relaxations for accurate evaluation of the ionic forces. Thus, the adopted computational protocol for gas phase optimizations is PBE0-D4/def2-TZVP. After geometry optimizations, no imaginary frequencies were found in the vibrational analysis. This confirms that all the relaxed geometries are true stationary points on the electronic ground state potential energy surface. We used effective core potentials (ECP) to describe the core electrons in the transition metal Ag atom. A typical ORCA keyword used in our input files was "!PBE0 D4 DEF2-TZVP RIJCOSX def2/J TIGHTSCF OPT."

ADDITIONAL DATA AND IMAGES

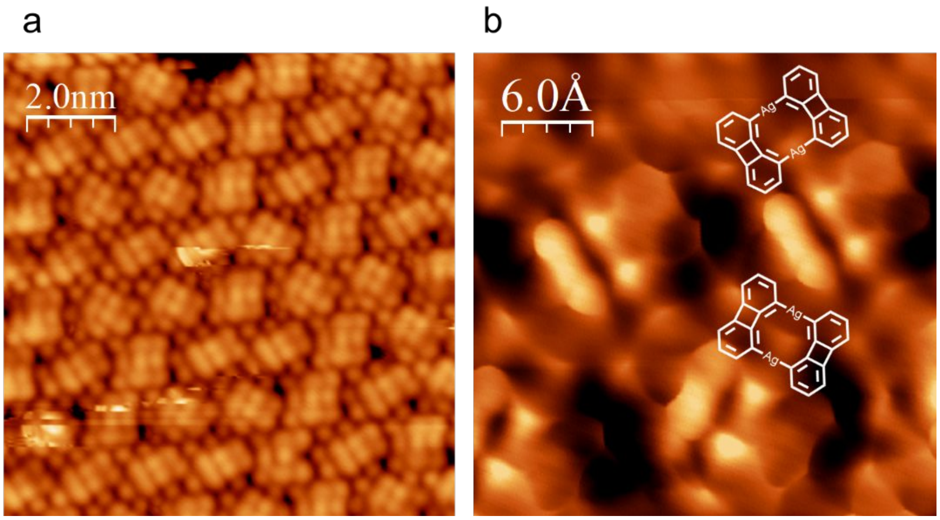

Figure S1. a) STM image upon deposition of **1** on Ag(111). (I=100 pA, U=-500 mV, 5 K) (b) Constant-height bond-resolving images of the **2-Ag** with a CO-tip (U=5 mV).

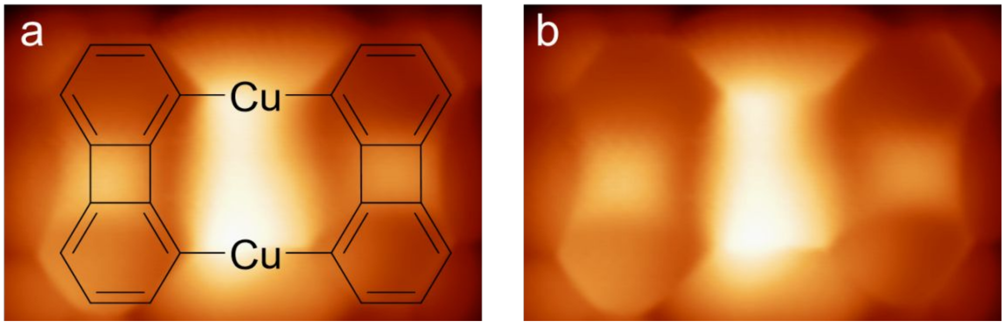

Figure S2. (a). Superimposed structure of **2-Cu** on STM image of main text Figure 1c and (b) STM image without the structure for comparison.

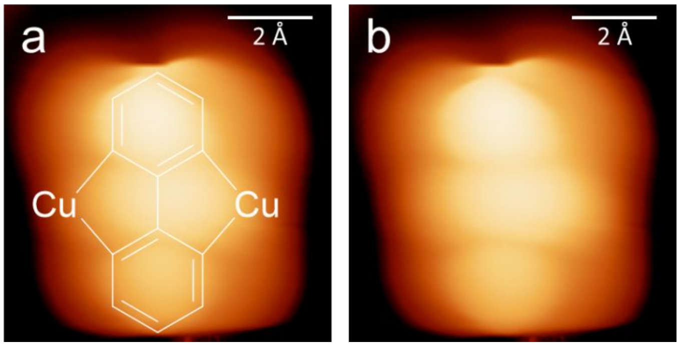

Figure S3. (a) Superimposed structure of **4** on STM image of main text Figure 1d and (b) STM image without the structure for comparison.

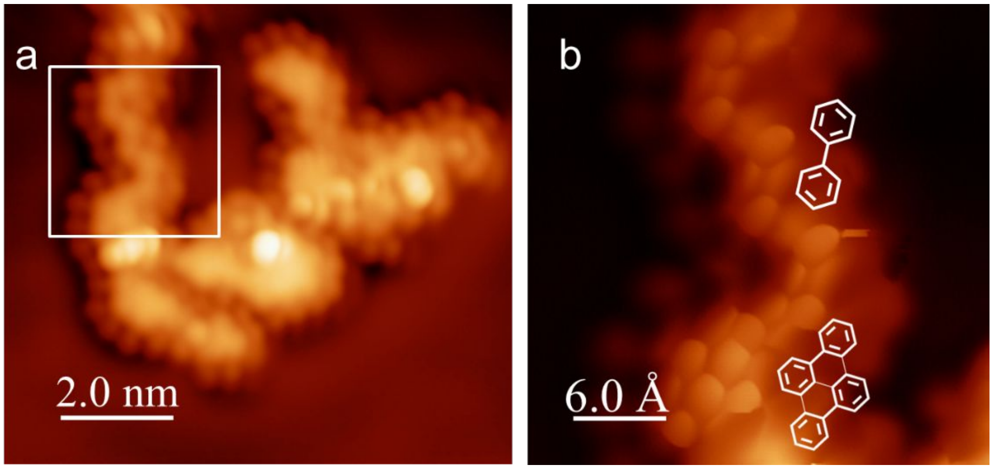

Figure S4. (a) Supplementary STM image upon annealing **2-Cu** to 300 °C (I=100 pA, U=-500 mV, 5K). (b) Bond-resolving image using a CO tip of the region indicated by the white box in (a) (U=5 mV).

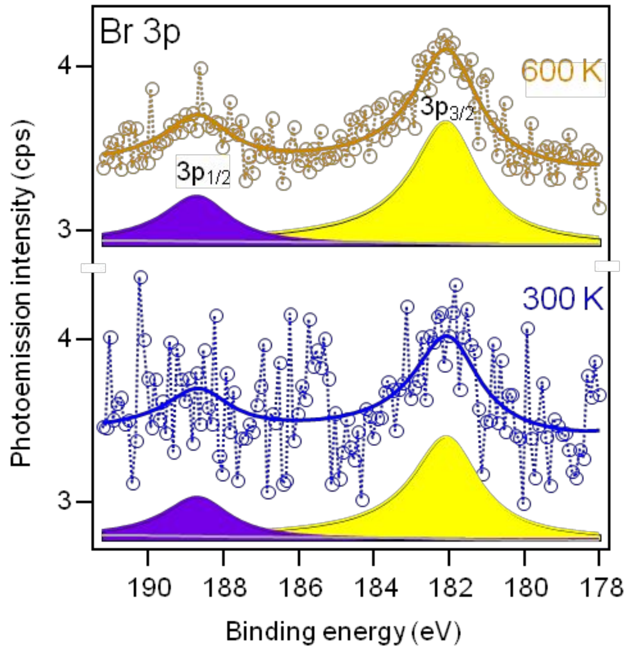

Figure S5. XPS spectra corresponding to the Br 3p core levels. No changes were seen upon annealing the sample to 600 K.

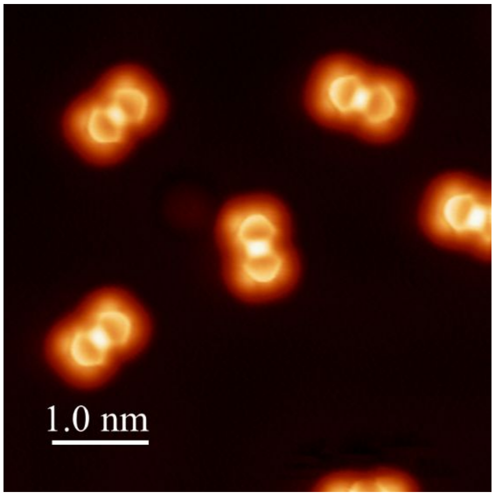

Figure S6. Constant height bond-resolving STM image of a sample of biphenylene left at room temperature for two days (U=5 mV, 5 K).

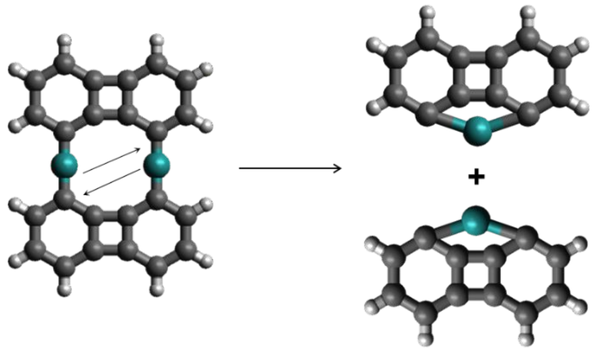

Figure S7. Reaction of homolytic decomposition of **2** to two mono-metallated units. We define BE = 2 E(mono-metalated monomer in the product) – E (**2** in the reactant).

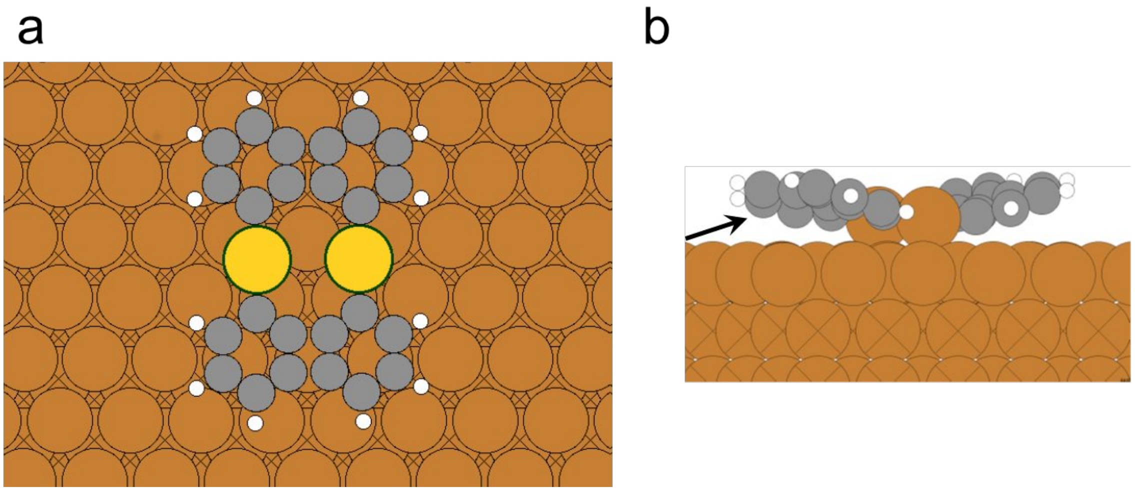

Figure S8. DFT-optimized geometry of **2-Cu** on Cu(111). Top (a) and side (b) views. Black arrows indicate the proposed attack of Cu adatom on the four-membered ring.

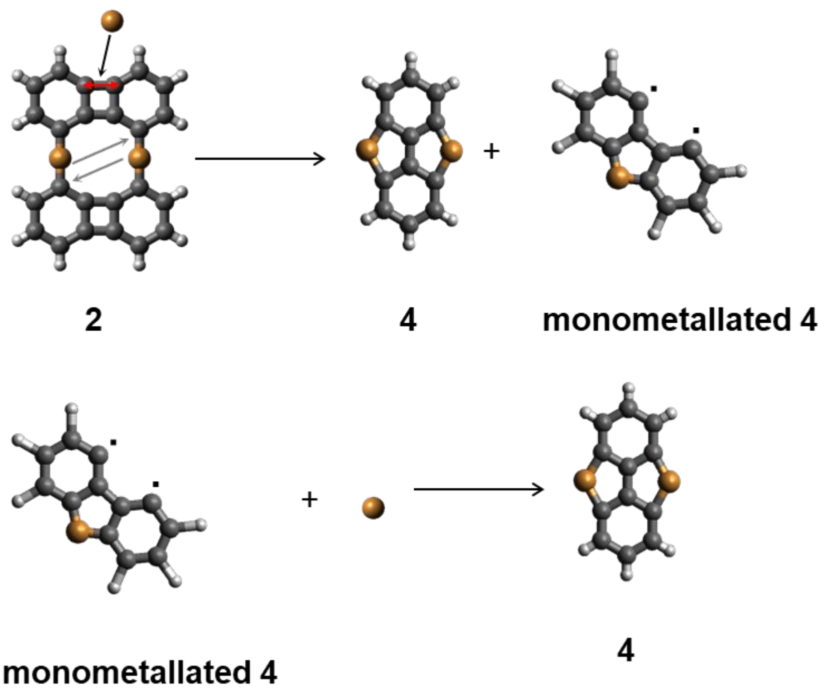

Figure S9. Illustration of the proposed mechanism for the formation of organocopper compound **4** from **2**. Orange spheres represent Cu atoms.

References

(1) Doniach, S.; Sunjic, M. Many-Electron Singularity in X-Ray Photoemission and X-Ray Line Spectra from Metals. *J. Phys. C: Solid State Phys.* **1970**, *3* (2), 285–291. https://doi.org/10.1088/0022-3719/3/2/010.

(2) Perdew, J. P.; Burke, K.; Ernzerhof, M. Generalized Gradient Approximation Made Simple. *Phys. Rev. Lett.* **1996**, *77* (18), 3865–3868. https://doi.org/10.1103/PhysRevLett.77.3865.

(3) Grimme, S.; Antony, J.; Ehrlich, S.; Krieg, H. A Consistent and Accurate *Ab Initio* Parametrization of Density Functional Dispersion Correction (DFT-D) for the 94 Elements H-Pu. *The Journal of Chemical Physics* **2010**, *132* (15), 154104. https://doi.org/10.1063/1.3382344.

(4) Hjorth Larsen, A.; Jørgen Mortensen, J.; Blomqvist, J.; Castelli, I. E.; Christensen, R.; Dułak, M.; Friis, J.; Groves, M. N.; Hammer, B.; Hargus, C.; Hermes, E. D.; Jennings, P. C.; Bjerre Jensen, P.; Kermode, J.; Kitchin, J. R.; Leonhard Kolsbjerg, E.; Kubal, J.; Kaasbjerg, K.; Lysgaard, S.; Bergmann Maronsson, J.; Maxson, T.; Olsen, T.; Pastewka, L.; Peterson, A.; Rostgaard, C.; Schiøtz, J.; Schütt, O.; Strange, M.; Thygesen, K. S.; Vegge, T.; Vilhelmsen, L.; Walter, M.; Zeng, Z.; Jacobsen, K. W. The Atomic Simulation Environment—a Python Library for Working with Atoms. *J. Phys.: Condens. Matter* **2017**, *29* (27), 273002. https://doi.org/10.1088/1361-648X/aa680e.

(5) Kresse, G.; Joubert, D. From Ultrasoft Pseudopotentials to the Projector Augmented-Wave Method. *Phys. Rev. B* **1999**, *59* (3), 1758–1775. https://doi.org/10.1103/PhysRevB.59.1758.

(6) Mortensen, J. J.; Hansen, L. B.; Jacobsen, K. W. Real-Space Grid Implementation of the Projector Augmented Wave Method. *Phys. Rev. B* **2005**, *71* (3), 035109. https://doi.org/10.1103/PhysRevB.71.035109.

(7) Enkovaara, J.; Rostgaard, C.; Mortensen, J. J.; Chen, J.; Dułak, M.; Ferrighi, L.; Gavnholt, J.; Glinsvad, C.; Haikola, V.; Hansen, H. A.; Kristoffersen, H. H.; Kuisma, M.; Larsen, A. H.; Lehtovaara, L.; Ljungberg, M.; Lopez-Acevedo, O.; Moses, P. G.; Ojanen, J.; Olsen, T.; Petzold, V.; Romero, N. A.; Stausholm-Møller, J.; Strange, M.; Tritsaris, G. A.; Vanin, M.; Walter, M.; Hammer, B.; Häkkinen, H.; Madsen, G. K. H.; Nieminen, R. M.; Nørskov, J. K.; Puska, M.; Rantala, T. T.; Schiøtz, J.; Thygesen, K. S.; Jacobsen, K. W. Electronic Structure Calculations with GPAW: A Real-Space Implementation of the Projector Augmented-Wave Method. *J. Phys.: Condens. Matter* **2010**, *22* (25), 253202. https://doi.org/10.1088/0953-8984/22/25/253202.

(8) Pérez Paz, A.; Rubio, A. Hydrated Alkali Atoms on Copper(111): A Density Functional Theory Study. *J. Phys. Chem. C* **2021**, *125* (7), 3868–3879. https://doi.org/10.1021/acs.jpcc.0c10061.

(9) Neese, F. The ORCA Program System. *WIREs Comput Mol Sci* **2012**, *2* (1), 73–78. https://doi.org/10.1002/wcms.81.

(10) Adamo, C.; Barone, V. Toward Reliable Density Functional Methods without Adjustable Parameters: The PBE0 Model. *The Journal of Chemical Physics* **1999**, *110* (13), 6158–6170. https://doi.org/10.1063/1.478522.

(11) Caldeweyher, E.; Bannwarth, C.; Grimme, S. Extension of the D3 Dispersion Coefficient Model. *The Journal of Chemical Physics* **2017**, *147* (3), 034112. https://doi.org/10.1063/1.4993215.

(12) Weigend, F.; Ahlrichs, R. Balanced Basis Sets of Split Valence, Triple Zeta Valence and Quadruple Zeta Valence Quality for H to Rn: Design and Assessment of Accuracy. *Phys. Chem. Chem. Phys.* **2005**, *7* (18), 3297. https://doi.org/10.1039/b508541a.
(13) Weigend, F. Accurate Coulomb-Fitting Basis Sets for H to Rn. *Phys. Chem. Chem. Phys.* **2006**, *8* (9), 1057. https://doi.org/10.1039/b515623h.
